# Optimal Lateral Displacement in Automatic Close-Range Photogrammetry

**DOI:** 10.3390/s20216280

**Published:** 2020-11-04

**Authors:** Gabriele Guidi, Umair Shafqat Malik, Laura Loredana Micoli

**Affiliations:** Department of Mechanical Engineering, Politecnico di Milano, Via La Masa, 1, 20156 Milan, Italy; umairshafqat.malik@polimi.it (U.S.M.); laura.micoli@polimi.it (L.L.M.)

**Keywords:** photogrammetry, computer vision, SfM/IM, image overlap, 3D data uncertainty, distance/baseline ratio, museum digitization, digital heritage

## Abstract

Based on the use of automatic photogrammetry, different researchers made evident that the level of overlap between adjacent photographs directly affects the uncertainty of the 3D dense cloud originated by the Structure from Motion/Image Matching (SfM/IM) process. The purpose of this study was to investigate if, in the case of a convergent shooting typical of close-range photogrammetry, an optimal lateral displacement of the camera for minimizing the 3D data uncertainty could be identified. We examined five different test objects made of rock, differing in terms of stone type and visual appearance. First, an accurate reference data set was generated by acquiring each object with an active range device, based on pattern projection (*σ_z_* = 18 µm). Then, each object was 3D-captured with photogrammetry, using a set of images taken radially, with the camera pointing to the center of the specimen. The camera–object minimum distance was kept at 200 mm during the shooting, and the angular displacement was as small as π/60. We generated several dense clouds by sampling the original redundant sequence at angular displacements (*n*π/60, *n* = 1, 2, … 8). Each 3D cloud was then compared with the reference, implementing an accurate scaling protocol to minimize systematic errors. The residual standard deviation of error made consistently evident a range of angular displacements among images that appear to be optimal for reducing the measurement uncertainty, independent of each specimen shape, material, and texture. Such a result provides guidance about how best to arrange the cameras’ geometry for 3D digitization of a stone cultural heritage artifact with several convergent shots. The photogrammetric tool used in the experiments was Agisoft Metashape.

## 1. Introduction

Automatic photogrammetry based on Structure from Motion and Image Matching (SfM/IM) is a game-changer in many fields where 3D digitization is needed. The fields of application are very different, ranging from industrial applications [1], deformation monitoring of structures and bridges [2], forestry assessment [3], forensics [4], medicine [5], zooarchaeology [6], paleontology [7], special effects for cinema [8], and cultural heritage documentation [9], to cite a few.

In the latter case, photogrammetry is particularly beneficial for the simplicity of the 3D acquisition and modeling process, the relatively low cost of the associated equipment compared to dedicated technologies like 3D laser scanners, and the possibility of extracting both the shape and texture of an object/scene from the same set of images. Those features involve a shortening of the time for creating a 3D model from the survey data, which could be several times shorter than using an active device [10], allowing a consequent reduction of costs, which is particularly critical in a low budget context, as that of digital cultural heritage.

For these reasons, SfM/IM is the methodology that has made possible the various initiatives of massive 3D digitization in museums [10,11,12,13] and heritage sites [14,15,16,17], which paved the way for broad digital access to heritage information that is otherwise impossible to gather, without a physical visit to the heritage venue in question.

### 1.1. Camera Orientation Factors Affecting Photogrammetric 3D Data in General

In all these fields, it was demonstrated that the quality of the captured 3D data in terms of measurement errors was related to the Ground Sampling Distance (GSD). This term originated in the Remote Sensing domain, with specific reference to aerial photogrammetry, where the target was quasi-planar, and the camera sensor plane was parallel to the target. The GSD was intended as the spacing between two adjacent pixels projected over the target surface [18], and for that reason remained constant in any area of the image. In close-range photogrammetry, especially when performed on cultural heritage artifacts such as sculptures, vases, and other museum objects, the target surface could be very far from a plane, therefore, the projection of the sensor’s pixel grid over the target gives a variable GSD with a minimum at the minimal target-camera distance. In any case, according to the literature, the smaller the GSD, the smaller the error [19,20].

In addition to this factor, the literature also reports the influence of the so-called baseline/distance ratio. In other words, when several images with the same GSD aim at the same feature from two different points of view, the measurement error affecting the estimation of the feature’s 3D coordinates also depends on the ratio between the displacement of the observation points (i.e., the baseline) and their average distance from the feature.

According to this view, considering a coordinate system centered in one of the two cameras where x and y identify the plane of the sensor and *z* the optical axis, we could express the standard deviation of error along *z* as [19]:

(1) $$\;\sigma_{z}=\;\frac{h^{2}}{b\cdot c}\sigma_{p}$$

where the meaning of the parameters is:

*h*  average distance between the two cameras and the feature (a graphical representation of this is shown in Figure 6.12 of [19] on page 511);
*b*  baseline;
*c*  principal distance (i.e., focal length of the lens plus additional shift to achieve sharp focus);
*σ_p_*  error affecting the estimation of the point over the camera image plane.

According to this relationship, fixing all the other parameters, the larger the baseline, the lower would be the error. However, it is also clear that the larger the baseline, the more difficult would be the identification of the feature on the two projections. Therefore, by distancing the two points of view too far, the result would be an increase of uncertainty in the positioning of the same feature on two different projections. This increased uncertainty on the plane of the sensor would then be projected in the 3D space, involving an increase of uncertainty in the estimation of the feature’s 3D coordinates.

These two opposite requirements led to the definition of the so-called 3 × 3 rules for simple photogrammetric documentation of architecture [21] in the 1990s, updated in 2013 [22], and adopted as a guideline by the “International Committee of Architectural Photogrammetry” (CIPA). According to these rules, the optimal baseline/distance ratio in the normal case (i.e., optical axes of the camera parallel to each other) ranged from 1:4 to 1:15 and from 1:1 to 1:15 in the convergent case. In any case the minimal suggested image overlap was 60%. The reason that the overlap and the baseline/distance ratio were mentioned as separate issues was that lenses with different focal length give different angles of view, also providing different overlap between the adjacent shots in correspondence to the same baseline/distance ratio.

The book by Luhmann et al. mentioned above [19] presents the same relationship (1) with a different formulation, trying to make evident the separate influence of the scale of the objects in the photogram and the mutual inclination of the cameras:

(2) $$\;\sigma_{z}=\;q\cdot m\cdot \sigma_{p}$$

where

*q = h/b*  represents the distance/baseline ratio or “design factor”, associated with the angle between the two lines of sight (i.e., the reciprocal of the baseline/distance ratio mentioned above);
*m = h/c*  represents the magnification factor, a different way of indicating the scale of the object on the photogram. Once the number of megapixels featuring the sensor is available, this is the same kind of information provided by the GSD in aerial photogrammetry.

According to this source, “practical values for the design factor *q* vary between 0.4–0.8 for excellent imaging configurations” in the case of convergent shooting all around the object. No numerical correspondence is associated with the idea of “excellent;” however, it is reasonable to interpret this as a configuration, leading to the minimization of measurement uncertainty. In any case, these configurations, leading to values below 1.0, indicate a baseline larger than the average distance of the camera from the target. Such a situation imply that the same 3D feature is observed from points of view very distant apart to each other. In this way, the corresponding 2D features originated by its projection on the sensor plane, might differ significantly.

By using the reciprocal of the numbers provided by the CIPA 3 × 3 rules, we can see that, in this case, the optimal *q* would range from 1 to 15, representing a much lower inclination of the camera’s lines of sight with respect to the “excellent imaging configurations” suggested by [19]. In this case, the baseline ranges from a value equal to the average distance of the camera from the target (*q* = 1), to a value much smaller than that (*q* = 15), in all cases with a span of more than one order of magnitude.

In both cases, the aspect related to the recognizability of the feature on the image are not raised. We think, instead, that this is a crucial point that deserves to be better analyzed.

### 1.2. Camera Orientation Factors Affecting Specifically SfM/IM 3D Data

When we move from traditional to automatic photogrammetry, identifying corresponding points on different images is no longer a manual activity. What makes automatic the photogrammetric process in SfM/IM is using a procedure that extracts a set of features from each image, which allows the more likely features to be identified without the intervention of a human operator. Algorithms such as the “Scale Invariant Feature Transform” (SIFT) [23], its approximated version “Speed up Robust Feature” (SURF) [24], the low complexity “Binary Robust Independent Elementary Features” (BRIEF) [25], or similar ones, are capable of associating an identifier, based on local luminance gradients, with each group of pixels emerging from the background for their luminance contrast. This makes it possible to identify the same feature on different views of the same scene. Being associated with parameters that are not necessarily univocal in the image, such correspondence is usually refined by algorithms that estimate the camera roto-translation from one position to another, determined as the mean pose estimation associated with the globality of the corresponding features, and identify which of the correspondences is too far from such behavior. The latter process is carried out by algorithms conceived for identifying outliers from a set of observed data such as “Random sample consensus” (RANSAC) [26], its evolution MLESAC [27], or other similar outlier removal processes [28]. This phase works better if the lateral displacement among images is lower than that afforded in the manual case, because this maximizes the recognizability of correspondences [29]. Once the set of corresponding features among two or more images is reliable, a bundle adjustment process makes it possible to precisely calculate the exterior orientations of the different camera positions, and the interior orientation of the camera used for the shooting, including the principal point coordinates, principal distance, and the various distortion parameters (radial, affine, and tangential).

However, it is the last phase of the process, the Image Matching (IM), which plays a major role in determining the measurement uncertainty, overlapped on 3D data. In this phase, image patches of any oriented image are matched with the most likely patches of a corresponding image, along the epipolar lines. Such a process might work on image couples, if stereo matching is implemented [30], or on several other images, in the case of multi-view stereo matching (MVS) [31]. This way of systematically extracting the dense cloud from the images affects the criteria for evaluating the optimal camera distribution in space, because the smaller the lateral displacement, the more similar would be the related projections of the same scene, and consequently the more accurate would be the matching process. Conversely, for large lateral displacements, the images would be more different from each other, and the more probable would be a wrong matching, increasing the probability of a wrong 3D estimation of the associated point. This result, when extended to a population of millions of points, would involve a general increase of 3D uncertainty overlapped over the actual 3D data [32].

### 1.3. Previous Works about Optimal Cameras Orientation in SfM/IM Photogrammetry

The presence of two opposed requirements—one for maintaining a low distance/baseline ratio *q* for making more robust the orientation of different cameras, the other for achieving a high *q* to maximize the SfM/IM process performance, led us to search for a compromise, which at least for close range photogrammetry, was never before quantitatively analyzed in terms of error versus *q*.

The works touching the influence of the distance-baseline ratio over the photogrammetric outcome mostly apply to aerial photogrammetry from drones, where the images are mainly nadiral (i.e., with the cameras’ optical axes orthogonal to the terrain and parallel to each other), where the effects of lateral displacement for a given focal length were evaluated globally in terms of image overlap. According to a traditional vision, the overlap between adjacent images in the aerial strips was set to 60%, as forward overlap (along-track), while the overlap between adjacent images between strips was set to 20% as side overlap (across track) [33,34]. However, Haala and Rothermel in 2012 [35], analyzing a UAV survey by nadiral images taken with a zoom camera Canon Ixus 100 IS with the minimal zoom setting (f = 5.9 mm) at a height of about 115 m (GSD = 30 mm), tried to experiment larger overlap values. The overlap was 75% in the flight direction and 70% among adjacent flight lines. The authors noticed that considerable overlap and image redundancy allowed a very efficient elimination of erroneous matches, improving the reliability of the 3D point cloud.

Such an outcome was further detailed by research in the field of forest canopy survey, showing a comparison between LiDAR and photogrammetric 3D data of the same forest, in 2015, which was obtained applying SfM/IM with different conditions of altitude and overlap [36]. The LiDAR data were used as ground truth and the 3D clouds obtained with photogrammetry were compared against them. The Root Mean Square (RMS) error of the 3D data deviation from the reference was used as an index for estimating the measurement uncertainty of each photogrammetric setup. One of the photo sets was produced by a drone equipped with a Canon ELPH 520 HS, flying at a fixed height of 80 m (GSD = 33.6 mm), with different overlap levels. The study’s results showed that the uncertainty level progressively decreased from 7 m to 1 m, corresponding to overlaps ranging from to 60% to 96%. Considering the camera and lens reported, we could estimate that the 60% overlap provides *q* = 1.9, while the 90% case gives *q* = 19.4, a design factor very far from the theoretical optimal values.

A deeper exploration of this point appeared in 2018 by Ni et al. [37], again in a study oriented to the survey of vegetation from drone images. In this case, a Sony NEX 5T was used on a drone flying at about 300 m from the ground, using the wider angle supported by the camera’s standard zoom lens (f = 16 mm), implying a GSD = 86 mm. In this case, 79 images were taken, choosing a baseline among adjacent shots (29 m) in such a way that 10% could be added to the previous image, along the flight direction. The image overlap of the raw data set was, therefore, high (90%) but could be reduced in steps of 10%, by leaving out an appropriate number of images in the set. For example, leaving out one image every two shots reduced the overlap from 90% to 80%; if two shots were omitted, the overlap was reduced to 70%, etc. Even though the purpose of this study was to determine the average accuracy of the forest canopy estimation, one of the incidental results showed the RMS error of the photogrammetric data against the LiDAR reference at variable overlap levels. These results make evident a decrease of RMS error for smaller overlaps with a re-growth of the RMS error at the maximum overlap. This suggests a possible worsening of the process when the overlap is too high, but also an optimal value for overlap levels much higher than the traditional 60%. These general data were confirmed by a recent review study about photogrammetry of forests, stating that an optimal image overlap should be in general >80% [3].

Another paper exploring the effects of image overlap over the photogrammetric 3D data quality, also published in 2018, refers again to measurements in the field of vegetation assessment [38]. In contrast to the papers cited above, this study was based on a scaled experimental setup made to produce a stronger control of the camera positioning. This makes the arrangement closer to the close-range configuration than we would like to analyze in this study. The experimental set-up consisted of a structure (7.3 m × 1.7 m) built by two sets of aluminum sliding tracks, positioned horizontally above the text field. A camera holder attached to the structure was moved through timing belts by two stepper motors on the xy plane. The movement along the longer structure dimension simulated the flight direction, while the other movement made it possible to generate different image stripes parallel to each other. Three test objects were used—a parallelepiped (73 mm × 48 mm × 98 mm), a cylinder, and a mushroom-shaped object consisting of a hemisphere lying on a cylinder. The two latter shapes had sizes similar to that of the parallelepiped. They were previously measured with a caliper and 3D digitized with photogrammetry, in different overlap configurations, with a camera-ground distance of 1.2 m, forward overlap ranging from 75% to 95%, and lateral overlap from 67% to 90%. A parameter called “Power Of Unit” (POU), which considers simultaneously the image resolution and the longitudinal and lateral overlap, was used to compare the results. Among other different considerations, it was shown that “the average measurement errors dropped dramatically with the increase of POU” (i.e., with larger overlaps).

Moving from the small scale of a forest, to the large scale of micro-photogrammetry, the literature presents few works studying the metrological behavior of SfM/IM photogrammetry in condition of convergent axes, through the study of the so-called “Photogrammetric Scanning System with Rotary Table” (PSSRT) used for the 3D acquisition of small mechanical parts. Such systems are designed to return precise 3D digital models of objects a few millimeters long, which present complex surfaces and sub-millimeter features. The discussion around 3D data quality in this domain includes the modelization of the measurement uncertainty in micro stereo-vision systems [39], or the influence of software algorithms on photogrammetric uncertainty [40].

The latter paper by Lavecchia et al., aims at demonstrating the good performances of such a system implemented with an SLR camera Canon 40D with an APS-C sensor equipped with a Canon EF 50 mm f1.8 lens focused to infinity and a 36-mm Kenko extension tube. The test, also methodologically explained in [41], consisted of measuring a pyramidal metrological specimen with a 24 mm × 24 mm square base, 2.75 mm tall. The dense clouds originated by photogrammetry in different shooting conditions were compared with a ground truth gathered by measuring the same object with a conoscopic laser scanning system. The photogrammetric output was generated from a single strip of images with the camera aiming at the test object from above, at a fixed tilt angle, and a lateral displacement of 5° or 20°, originating 72 or 18 images per strip, respectively. The experiments were carried out by testing two possible tilt angles—30° and 60°. 

Even if the aim of these experiments was not to evaluate error vs. angular displacement but rather the overall good performances of such scanning systems, in the experiments, it was possible to extract some clues about this point. By eliminating the tilt angle 30°, where the photogrammetric outcome was overly determined by the limited depth of field, from the paper data it was possible to estimate the camera-target distance (126 mm) and, therefore, the baseline associated to the 5° and 20° angular steps considered at the tilt angle of 60°. In this case, the paper data showed an average reduction of error passing from an angular step of 5° (q = 22.9 calculated on the paper data), to 20° (q = 5.7).

In order to make the above-mentioned data comparable to themselves and to the data reported in the experimental section of this paper, we calculated the design factor and other opto-geometrical parameters for the different situations described in the articles referenced above, reporting them in Table 1. It is worth highlighting that the so-called “forward” displacement in aerial photogrammetry corresponds to the “lateral” displacement between two adjacent images in the radial geometry.

### 1.4. Rationale of This Research

Several quantitative analyses were conducted in the field of aerial photogrammetry to establish how the SfM/IM approach influenced the optimal distance/baseline ratio, making it evident that an overlap much higher than that suggested by a traditional approach involved a reduction of measurement uncertainty. 

Although no similar systematic studies were done for close-range data sets with convergent lines of sight, results coming from the characterization of a photogrammetric system for 3D digitizing extremely small objects, suggest that *q* should not be as large as possible and an intermediate optimal value should exist.

This reinforces the observations of this paper’s authors in 3D digitization of Cultural Heritage, based on SfM/IM photogrammetry [10,11], and a preliminary study based on qualitative evidence emerging from such experiences [42], which makes evident a lack of scientific information about a possible optimal level of the *q* ratio in such applications. The purpose of this study is, therefore, to fill this gap by exploring with high granularity how the 3D measurement uncertainty changes in correspondence with different *q*, considering a small camera-target distance survey, similar to what happens in surveying stone sculptures or other museum artifacts.

## 2. Materials and Methods

This study defines a methodology to explore the influence of image lateral displacement by comparing the photogrammetric models of specific test objects with the accurate models produced by a metrology grade 3D device, based on pattern projection. The test objects were five stones with different geometrical structures and surface properties. These stones were first digitized by using a very accurate and precise pattern projection device, with a measurement uncertainty below 20 micrometers, to create the reference models. The same objects were then photographed under controlled conditions, giving a fixed and small angular rotation to the object, with respect to the camera, in consecutive images. Each image set gathered with the minimal angular displacement (i.e., made of the maximum number of images) was then aligned using the SfM process, in this way also obtaining a robust estimation of interior and exterior camera orientations. On the aligned images, several image matching processes were run, each time skipping one image every two, three, and so forth, up to seven images in every eight, in order to implement different distance/baseline ratio conditions. The resulting dense clouds were then accurately aligned and scaled against the reference model, in order to reduce the possible systematic error to a negligible component. The final step was to estimate the standard deviation of error of each cloud versus the corresponding reference model, for the purpose of estimating possible trends in 3D quality vs. angular rotation, the latter being directly related to the distance/baseline ratio.

### 2.1. Test Objects 

Five stones of different shapes and surface properties were carefully selected. This selection was made so as to simulate the surface properties of different materials that are mostly used for the manufacturing of sculptures, ranging from more to less optically cooperative surfaces.

**Figure 1:**
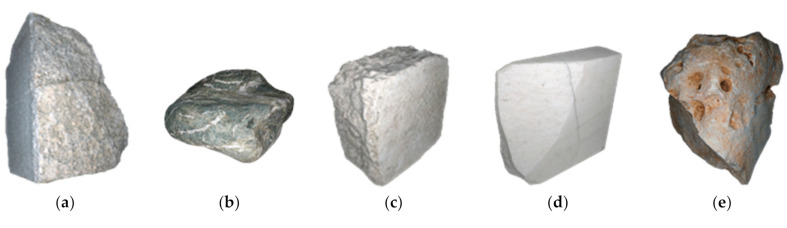
The five stone samples used as test objects in the experiments—(**a**) granite; (**b**) sedimentary rock; (**c**) limestone; (**d**) marble; and (**e**) sandstone.

The first stone was an irregular piece of granite with a sharp cut on a side. Its shape was, therefore, a combination of a flat planar surface with sharp edges on one side and irregular shapes on the other side. Its texture had a blend of little dark spots on the light background, which might favor the feature’s detection process (Figure 1a).

The second stone (Figure 1b) was a sedimentary rock smoothed by the water of a river. For this reason, it had a continuous smooth surface with round edges. The texture was a mix of prevailing uniform dark brown with whiteish veins that could be suitable for automatic feature extraction. 

The third sample (Figure 1c) was a roughly cuboid piece of limestone with irregular surfaces, opaque appearance, and a light specked texture that makes it very suitable to SfM photogrammetry.

The fourth specimen is a regular box-shaped slab of marble with a large chip of material lacking on a side (Figure 1d). The shape was, therefore, mostly regular, apart from the broken part that presents an irregular surface. The material in this case was optically non-cooperative, as demonstrated by tests with active 3D devices [43,44], due to its sponge-like microstructure, which cause light to be reflected not only by the exterior surface but also by several inner layers of material.

The fifth sample was a piece of sandstone with the most irregular shape among all objects of the set, including nearly flat surfaces, sharp-edged, rounded zones, and several surface cavities (Figure 1e). The feature detection process was highly affected in the parts where cavities were present. Except for these cavities, the shape and surface colors elsewhere on this object were very suitable for feature detection during the photogrammetric process.

### 2.2. Devices

**Figure 2:**
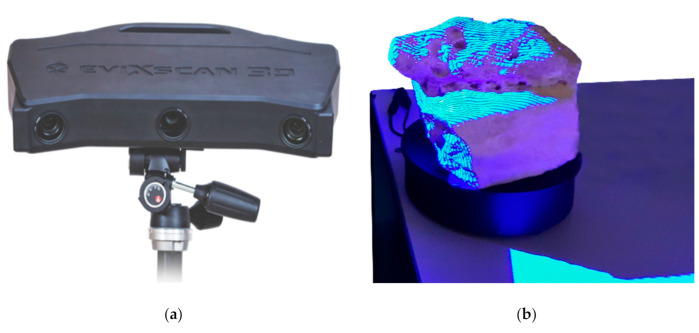
3D digitizer used for creating the high accuracy and high-resolution ground truth 3D model for evaluating the dense point clouds created by photogrammetry: (**a**) eviXscan 3D Heavy Duty Optima pattern projection device with one central projector an two 5M pixel cameras on the sides; and (**b**) the test object placed on the turn table attached with the device, while illuminated by one of the blue light stripe patterns.

**Figure 3:**
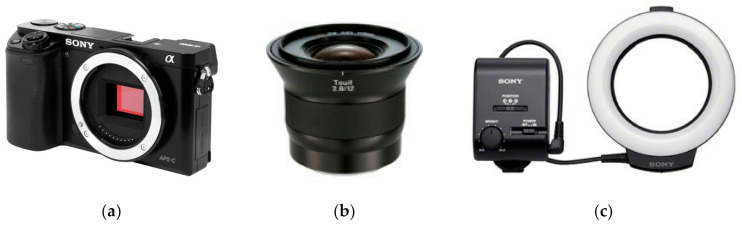
The photographic equipment used in the tests—(**a**) Sony a6000 camera body with CMOS sensor featuring 24 MPixels; (**b**) Zeiss Touit 12 mm f/2.8 lens; and (**c**) HVL-RL1 LED ring illuminator.

To create accurate reference models, a precision range sensing device was used. The eviXscan 3D Optima Heavy Duty was a blue light pattern projection device, from Evatronix S.A. equipped with one central blue light stripe projector and two lateral 5 Mpixel cameras (Figure 2a). With one shot, it generated a 5 Mpoint range map, reducing the effect of possible occlusions, thanks to the double camera. It achieved high density of 3D point clouds (up to 116 pt/mm^2^), corresponding to a lateral resolution better than 0.1 mm. The wavelength of blue light helped to limit the influence of ambient light on the measured data (Figure 3b). Furthermore, owing to the low diffraction of blue light, the resulting measurements were more accurate than those originating from the white-light patterns. The measurement accuracy provided by the manufacturer was up to 18 µm, evaluated in the factory through the procedure defined by the German meteorological standard VDI/VDE2634, part 2, for characterizing industrial full-frame devices by 3D digitizing certified ball-bars [45]. The measurement volume was 250 mm × 170 mm × 120 mm.

The device was mounted on a tripod and connected with a turntable, which could be controlled by entering a turning angle for each measurement into the device’s control software (eviXscan 3D Suite). In this way, all the range maps generated for an entire 360-degree turn could be pre-aligned automatically. The final alignment was then refined by using an Iterative Closed Point (ICP) algorithm implemented in the eviXscan 3D Suite, which makes it possible to estimate the standard deviation of error on the overlapping areas of various range maps.

The photographic equipment for this test was selected based on our previous experiences of photogrammetric survey in the most difficult situations for photogrammetry. The combination of camera and lens was the one that was used for digitizing the archeological sculptures placed near the walls, under the auspices of the Uffizi-Indiana University project [10].

As learnt from the photogrammetric survey during this massive 3D digitization of the sculptures, the placement of several sculptures in the museums did not support photography from sufficiently long distances. For such situations, a wide-angle lens is required to shoot images from short distances. Furthermore, there were several locations inside the museums where enough natural light was not present, especially inside the exhibition rooms. In these cases, to photograph the dark areas of sculptures, an artificial ring light was used in the project. The combination of camera sensor, lens, and ring light allowed us to take suitable images for photogrammetry, for a sensor-to-object distance as small as 20 cm, even in the absence of natural light. 

To simulate the actual difficult situations of photogrammetric survey in the field, but also for creating a controlled and repeatable lighting condition for all experiments, the same combination of camera and lens, coupled with the ring light was used for the tests presented in this study. The camera was a mirrorless Sony α6000, featuring a 24 megapixel APS-C CMOS sensor with an E-mount for interchangeable lenses (Figure 3a). It was coupled with a Zeiss Touit 12 mm f/2.8 Lens, which had one of the widest fields of view in APS-C format, among the commercially available lenses, allowing us to focus up to a very short camera-target distance (18 cm), which makes it ideal for accessing possible narrow spaces behind sculptures (Figure 3b). The LED ring illuminator HVL-RL1 was attached to the lens (Figure 3c). This light is designed for video recording; therefore, it provides continuous illumination in contrast with a flash. The continuous illumination feature ensures the proper functionality of the autofocus during the collection of the image set, even from the darker areas of the scenery, normalizing the illumination conditions on every shot. Although using a light attached to the camera could be questionable in photogrammetry, owing to the actual change of mutual object-light orientation for each shot, which might affect the appearance of each feature over the surface of the surveyed object, previous experimental work demonstrated that for small displacements of the point of view, such influence is negligible [10].

To avoid any possible oscillation during the shoot, the camera was equipped with the “Smart Remote Control” function, which makes it possible to connect the device to a tablet or a smartphone using the app “Sony Play Memories”. Through the Wi-Fi connection, the viewfinder and the controls over shutter speed, aperture, and ISO sensitivity were remotely available on the connected portable device. The focusing could also be remotely set on a specific point by tapping on the desired area over the remote viewfinder.

### 2.3. Reference 3D Models

To create the accurate reference models to be compared with the point cloud generated by photogrammetry, the pattern projection device described in the previous section was used. Several range maps were captured by the eviXscan 3D Suite. Thanks to the turntable controlled by the software, the range maps were automatically pre-aligned for a complete rotation of the test objects, from different points of view.

These stripes of aligned 3D images were then manually pre-aligned, each to the others, and all the range maps covering the complete surface of the test objects were then globally registered through the ICP algorithm embedded in the software. The residual RMS error among different aligned range maps was evaluated over the overlap areas, providing values ranging from 10 to 20 µm, coherent with the accuracy declared by the manufacturer [46]. Considering that the uncertainty of the photogrammetric point clouds that we would study in this study was generally above 100 µm, the range device data result affected by errors were at least five times lower than those provided by photogrammetry. This made the active device 3D data suitable as ground truth, for studying the effect of camera orientations vs. the photogrammetric outcome.

Finally, the aligned range maps were merged in the form of a mesh, applying the 3D data interpolation algorithm implemented by the Evixscan 3D. Here, all points of the 3D cloud were also nodes of the mesh. In this way, no smoothing of the acquired data occurred, therefore maintaining, the accuracy of the raw 3D data. Due to the high density of the device’s measurement points, the resulting mesh had a large number of faces (on the order of tens of millions) to be used as reference for comparison with the point clouds from photogrammetry, with many fewer points (on order of 2–3 million).

Therefore, both the metrological properties of the scanned data and their spatial density were suitable for being used as a reference for comparison with the photogrammetric 3D clouds.

The meshes for all test objects were exported in STL format. Before carrying out the comparison, all meshes were topologically cleaned and oriented in Meshlab, the well-known open source mesh processing software developed by the Visual Computing Lab at ISTI-CNR, Italy [47]. The cleaning process was performed to remove duplicate vertices and faces, non-manifold edges and vertices, and zero area faces, in order to remove any possible systematic error in the comparison. 

**Figure 4:**
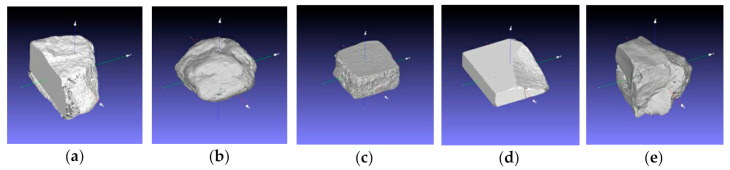
Meshes of the object described in Section 2.1 created by active 3D digitization with the pattern projection range device described in Section 2.2. Each 3D model was translated with the origin of the reference system on its barycenter, with xy representing the horizontal plane: (**a**–**e**) correspond to the specimen shown in Figure 4.

The orientation was a translation of the coordinate system to the barycenter of each mesh, and a rotation for having an ordered representation of the 3D models, as shown in Figure 4. Such meshes allowed us to precisely measure the bounding box of each reference sample, as reported in Table 2. The numbers make evident that all objects fall within the same order of magnitude, with a diagonal roughly ranging from 300 to 400 mm. This feature makes them suitable for a comparison, where the 3D capturing method remains the same (SfM photogrammetry), the opto-geometrical conditions are controlled, while the material and texture change from case to case.

### 2.4. Methodology for Generating the Photogrammetric Dense Cloud

#### 2.4.1. Experimental Set-Up

**Figure 5:**
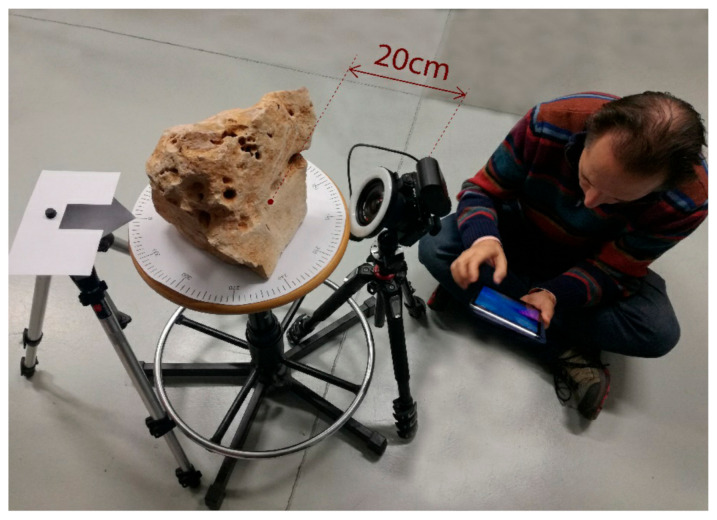
Experimental set-up for generating image sets at controlled angular steps, involving a small, fixed lateral displacement. The distance between the camera and the object is roughly evaluated with a tape meter from the sensor plane to the closest point of the surface in front of the camera.

The rig for performing photogrammetric survey was designed in a simple but effective way. It was composed of three components (Figure 5)—(i) a manually rotating platform at the center of which the test object was placed, which allowed a minimum angular rotation of π/60; (ii) a first tripod with an arrowhead mounted on the top to point at the angular scale for controlling the rotation; and (iii) a second tripod holding the camera equipped with the 12 mm lens and a ring illuminator, described in the previous section. 

The distance between the nearest point of focus on the object and the camera was kept at approximately 200 mm, as allowed by the combination of camera, lens, and ring illuminator, described in the previous section. By using this setting, it was possible to photograph the test objects with a minimal lateral displacement, given by the distance between the sensor and the center of rotation, multiplied by the angular step Δ*θ* = π/60. Considering the size of each object to be roughly represented by the diagonal of each bounding box reported in Table 2, we could approximately estimate the distance between the camera and the rotation center as:

(3) $$r=\;\frac{d}{2}+200\;mm$$

The minimal distance between adjacent shots (i.e., the minimal baseline of the photogrammetric image set), was therefore:

(4) $$b=r\frac{\pi }{60}\;$$

Therefore, by evaluating the camera-target distance fixed at 200 mm, we could calculate the maximum *q* factor considered in these experiments, as a function of the object size d:

(5) $$q=\frac{60}{\pi \;\left(\frac{d}{400}+1\right)}\;$$

The following photogrammetric process would consider different image subsets of the denser one by skipping n − 1 images every n, with n ranging from 1 to 8. This would involve an angular displacement Δ*θ = n*π/60, and consequently *b(n)* would be:

(6) $$b\;\left(n\right)=r\frac{n\pi }{60}\;$$

Consequently, the *q* factor in correspondence of different skip levels n would be:

(7) $$q\left(n\right)=\frac{60}{n\pi \;\left(\frac{d}{400}+1\right)}\;$$

#### 2.4.2. Image Capturing Protocols

The images were acquired in very controlled conditions, with the camera set on “A” mode, in order to maintain the same aperture on each image (F16). This allowed to set the depth of field at a level compliant to the shapes of the samples. In this condition, setting the focusing at 25 cm, the depth of field ranged from 16.4 cm to 53.1 cm, suitable to have all of the imaged surfaces in all 5 samples, in focus. In addition, a strict control over the aperture allows to limit the large influence of the aperture on the radial distortion changes, improving the calibration following outcome.

The ISO level was fixed at 400 for each data set, except for object (e), which was acquired at ISO 100.

The lighting was provided by the ring illuminator shown in Figure 3 and Figure 5. Its intensity was regulated in order to have a uniform distribution of light through the entire framed area. The setting was left the same for each one of the 5 photogrammetric campaigns.

Since texture and reflectivity of the objects were different, the only parameter that the camera could set automatically was the shooting time for avoiding under/over expositions and the autofocus, which, considering the constrained shooting conditions and the regularity of the different objects, gave focusing variations in order of few centimeters.

In all shooting sessions, the images were saved using the Sony RAW image format (ARW). This made it possible to maintain the whole 12-bit dynamic range captured by the camera sensor, allowing a possible image balancing in a subsequent phase in case of nonuniform light distribution throughout the photogram. However, due to the very controlled conditions of the shooting phase, such post-processing turned out to be unnecessary, and the images were directly converted in jpeg (8-bit) for the following photogrammetric processing. The conversion was carried out with the commercial software package Lightroom (Adobe) at the lowest compression level allowed.

**Figure 6:**
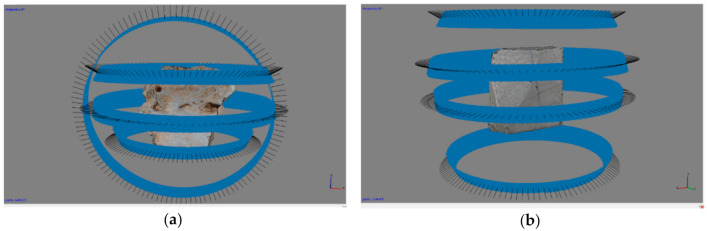
The protocol for capturing photos of the test objects—(**a**) used for test objects “a” and “e”; and (**b**) used for test objects “b”, “c”, and “d”.

For each object, four blocks of 120 images were taken all around the test object to cover the entire 2π rotation. As shown in Figure 6, two different camera distributions around the object were used.

The first configuration (Figure 6a) was composed of three horizontal image blocks taken with the point of view at three different heights and the camera aiming at the center of object. To connect the first three blocks, a block of images with projection centers on a plane orthogonal to the planes defined by the projection centers of the other three blocks was used. This latter block was obtained by just rotating the specimen on the rotary table of 90° around a horizontal axis, without changing anything in the geometrical relationship between the camera and the rotary table. In all of these blocks, the minimum distance of 20 cm between the point of focus on the object and camera sensor was maintained. Based on our experience, such a protocol for image capturing is preferable. Nevertheless, in actual conditions, this 90° rotation does not always allow to maintain a stable positioning of the sample over the rotating stand. In such cases, another configuration was employed, where all images were captured in blocks at different heights with a higher overlap among the different blocks, but without any dataset defining a plane orthogonal to the other three (Figure 6b).

Although the former would be theoretically preferable, the latter also represents a reliable shooting protocol, extensively tested in digitizing museum objects located in contexts where moving all around the object is not possible.

#### 2.4.3. Image Processing and Photogrammetry

Although several commercial and open source solutions are now available for implementing the SfM/IM pipeline, in this study, we decided to use one of the software packages used more in museum and archaeological applications and widely reported in the literature: Agisoft Metashape Pro (St. Petersburg, Russia). The software package (v. 1.5.5) was run on a Windows desktop PC configured with an Intel i7-6800k CPU (8 cores), 64GB RAM, 512 GB SSD hard drive, equipped with a NVIDIA GeForce 1080 GPU with 4GB RAM.

The photo shoot of each object produced four 120 image blocks for a total of 480 images per object. Even if there was a black background, all images were manually masked before the feature extraction step to prevent possible steady features in the scenes from interfering with the SfM process. In this way, only the features extracted over the stone surface were used as tie points for calculating the mutual orientation of the images and the camera calibration parameters.

For each test object, the related 480 masked images were aligned and used as a starting point for the point cloud generation. The Metashape Pro software supports setting several parameters for aligning the images, including the following critical parameters: “Accuracy,” “Tie points limit,” and “Key point limit.” “Accuracy” defines the sampling level of the image for extracting features. When this parameter is “High”, the software works with the photos of the original size, “Medium” causes image downscaling by a factor of 4 (2 times by each side), and “Low” involves a downscale by a factor of 16, and Lowest value means further downscaling by 4 times more. The “Key point limit” represents the upper limit of the detected features to be considered on each image during the feature extraction step, and the “Tie point limit” represents the upper limit of matching features for every image [48]. 

In all experiments, the aligning accuracy was set at “High” to perform the feature detection process on the original photos without downscaling them. The key points and tie points limits were set to 40,000 and 10,000, respectively, and the feature detection process was constrained by the previously created masks.

The camera calibration was carried out on the full set of 480 images within the alignment process, using all available tie points identified in the unmasked areas, whose number was higher than 250 k for all considered specimens. This large number of tie points allowed to run a bundle adjustment, obtaining a rather accurate self-calibration made of the typical 10 parameters of the Brown model [49]—principal distance, 2 coordinates of the principal point, 3 polynomial coefficient of the radial distortion, 2 coefficient for the affine (or axial skew) distortion, and 2 for the tangential (or decentering) distortion. In addition to such 10 parameters, the same process calculated the 480 sets of 6 parameters, identifying the position (*x_i_*, *y_i_*, *z_i_*) and orientation (*ω_i_*, *φ_i_*, *κ_i_*) of each shot, with *i* = 1, 2, …, 480.

However, before proceeding with the following step, we checked the quality of those parameters for avoiding errors generated by possible wrong or inaccurate features generated in this phase. The feature extraction algorithm implemented in Agisoft Metashape is not publicly disclosed, as it is an industrial product. The only available information can be found on the web, in the Agisoft forum (https://www.agisoft.com/forum/index.php?topic=89.0), where a member of the technical support stated in 2011 that the feature extraction process “is similar to the well-known SIFT approach, but uses different algorithms for a little bit higher alignment quality.”

Since we do not know more than this, we used the tie points’ reprojection error for checking the quality of the calibration and orientation steps, independent of the feature extraction details. Therefore, our processing protocol involved the following steps:

1.  Run the alignment procedure on the full set of 480 jpg images captured as described above;
2.  Check the reprojection error on the resulting tie points. If below 0.5 pixels, stop here, otherwise proceed with the next step;
3.  Manually delete the tie points, providing more than 0.5 pixels of reprojection error;
4.  Rerun the bundle adjustment step on the cleaned set of tie points and go back to step 2.

The number of good tie points survived to this cleaning was in the order of 250 k for all objects, except for specimen d. In this case, the marble surface was less optically cooperative and featured, but still, the remaining tie points were about 200 k.

The interior and exterior orientations were kept for all the following processing steps, for fixing as much parameters as possible in this multiparametric problem, and in this way checking the sole influence of the different geometrical settings on the dense cloud assessment.

The IM phase for generating the dense clouds was repeated several times, starting from the same alignments and calibration, by using all images at the first step and gradually skipping images for subsequent IMs—one taken and one skipped in the second run, one taken and two skipped in the third, and so forth, until seven images were skipped after every aligned image in the last dense cloud generation. As the minimum angular displacement between consecutive images was set to be π/60, the resulting eight IMs included images with an angular displacement of Δ*θ = n*π/60, where n is the “skipping level” ranging from 1 to 8. In this phase, one of the main adjustable parameters is the “Quality,” which defines the size of the matching patch in the IM process. It could range from 1 × 1 pixel (ultra-high) to 16 × 16 pixels (lowest). Apart from the misleading choice of nomenclature, for this parameter, which essentially defines the density of the 3D dense cloud rather than its quality (which tends to be better for larger matched patches), our choice was to use the 4 × 4 matching patch, labeled by the software as “Medium.” That is, the default value proposed by the procedure and based on our previous experiences, gave the best tradeoff between cloud density, 3D uncertainty, and processing time. The other parameter that was optimized for this process was the “Depth Filtering,” which we chose to be “Aggressive”, in order to sort out most of the outliers. 

No scaling based on GCPs or reference distances was performed at this stage. The resulting dense clouds were exported in the form of a text file with six columns representing location (*x*, *y*, *z*), color (RGB), and normals (*N_x_*, *N_y_*, *N_z_*) of each acquired point.

### 2.5. Photogrammetric Data Scaling and Comparison with the Reference Mesh

These two last steps of the process involved the use of the open source software CloudCompare (www.cloudcompare.org).

As the point clouds resulting from the process described above were not scaled, before starting the comparison, all point clouds were roughly scaled on the basis of the approximated measurements taken on the mesh model created by the pattern projection device. These coarsely scaled models were then manually aligned with the reference mesh. Afterwards, the ICP algorithm embedded in CloudCompare was run twice for 50,000 points, first by only roto-translating the point cloud to the reference, and a second time, for finely adjusting the scale and orientation of the point cloud, with respect to the reference mesh.

The residual deviation of the 3D coordinates gathered with photogrammetry from the reference mesh was then statistically analyzed to calculate RMS error, mean error, and error histogram. We made sure that the scaling/orientation step was iterated until the mean value was less than 10 µm, for each dense cloud. This allowed us to confirm that the alignment and scaling process were performed correctly, in this way not influencing the random error estimation with a systematic component.

This process was repeated for all 8 point clouds of each one of the 5 test objects, for a total of 40 measurements of point-cloud vs. reference mesh. The results of such comparisons are reported in the next section.

## 3. Results

After the photoshoot, five sets of 480 extremely overlapped jpeg images were aligned in around 2 and one-half hours on the workstation mentioned above. All images were properly aligned for each of the five test objects.

The alignment process yielded more than 200k tie points in all cases. The protocol used involved the elimination of possible tie points with a reprojection error above 0.45 pixels and the optimization of the camera parameters with the remaining high-quality tie point. In this way, the reprojection error of tie points was better than 0.5 pixels for all five test objects.

The following dense cloud generation proceeded, as described in the previous section. Eight subsets of the 480 aligned images were processed, selecting all images, skipping one image every two, two every three, and so on, up to one image taken, and seven skipped. The consequent angular displacement between adjacent images expressed in radians was Δ*θ = n*π/60; *n* = 1, 2, ..., 8, corresponding to 3°, 6°, … 24°.

The corresponding image sets were, therefore, made by a decreasing the number of images, ranging from 480 for the first set to 480/8 = 60 for the last one, as reported in detail in Table 3.

Given the size of the objects and the related image distribution in space, these settings provided more than 2 million points for each of the 40 dense clouds created in this way (8 IMs at different lateral displacements replicated on all 5 objects).

**Figure 7:**
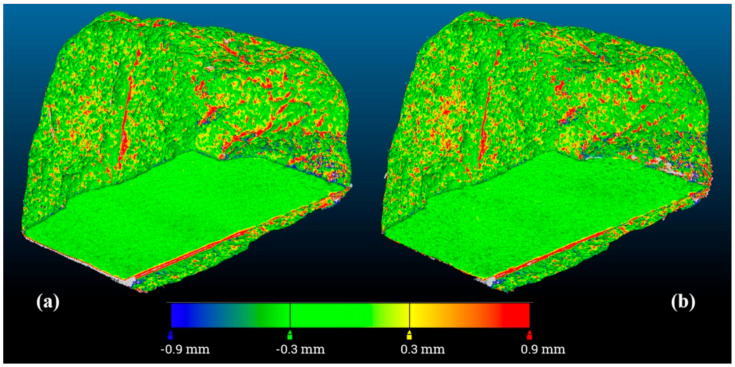
Example of color-coded map of deviations between the reference mesh and the photogrammetric dense cloud for the specimen a: (**a**) point cloud generated with all images of the block (480 images); (**b**) point cloud generated with 1 image every 8 (60 images).

Once scaled, each 3D point clouds compared against the reference mesh gave a residual deviation like that represented for example in Figure 7 for the specimen (a).

In this figure, the uniform distribution of the random error as well the absence of systematic errors such as those caused by a wrong alignment, which would give a large and uniform color variation from one side to the other of the map, was evident. The only systematic errors were those in presence of abrupt shape changes like in the corners and in the wrinkled surfaces. Here, the higher resolution of the 3D active device allowed us to capture most of the details, while the lower resolution of the photogrammetric dense cloud missed them. 

**Figure 8:**
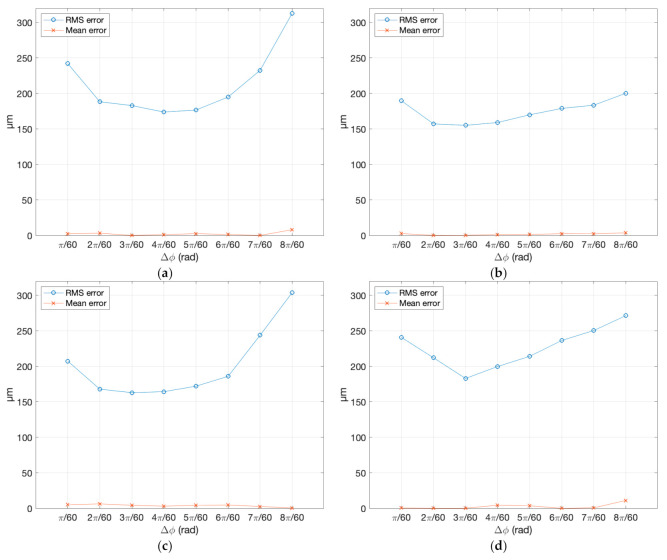

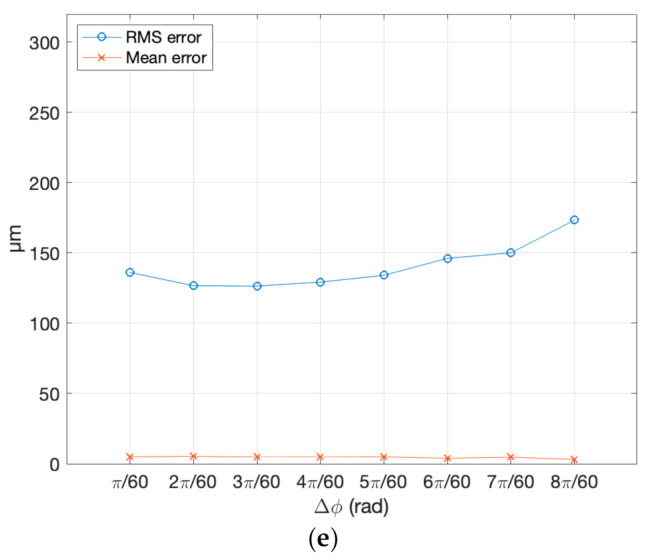
RMS error and mean error obtained by comparing eight different dense clouds created by various angular displacements with the reference mesh: (**a**) Granite sample; (**b**) sedimentary rock sample; (**c**) limestone sample; (**d**) marble sample; and (**e**) sandstone sample.

The analysis of all 40 dense clouds against the reference gave the values reported in Table 4. For the sake of clarity, these values representing the standard deviation of error of the 3D points created by the photogrammetric process with respect to the reference mesh, are plotted in Figure 8. The figure shows the behavior of the RMS and the corresponding mean error, in correspondence to different angular displacement between one image and the adjacent one, for all five samples chosen for the experiment.

In order to make these results comparable with the data reported in the literature, we needed to plot them against the distance/baseline ratio *q* that was used as common reference, for analyzing the conclusions reached in other studies. The quality factor *q* corresponding to the different angular displacements could be calculated with Equation (7), by approximating the size of each object with the diagonal of the related bounding box reported in Table 2. The *q* calculated for all 40 combinations of object size and angular displacement are reported in Table 5.

Owing to the similarity in size of the different samples, the related values were nearly identical, except for the smaller baseline (Δ*θ* = π/60) where *q* ranged from 9.38 associated with the larger object (sample 2) to 10.82 for the smaller one (sample 3). In this case, the average value across samples was 9.92. Such values allowed us to plot the RMS error behaviors, shown in Figure 8 in correspondence of Δ*θ* for each sample singularly, together with the residual mean deviation.

**Figure 9:**
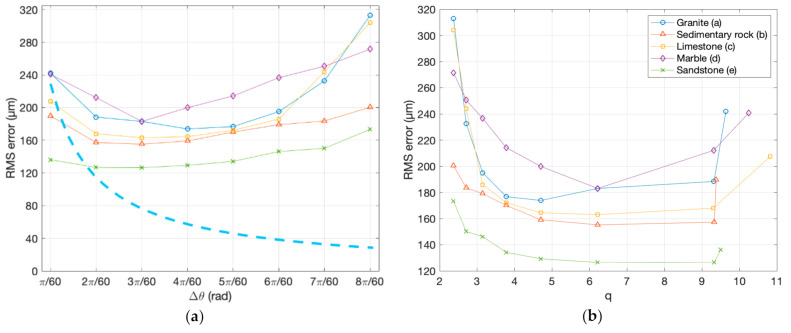
RMS errors of the five samples represented in different ways: (**a**) The five trends of Figure 4 vs. the angular displacement showing the theoretical value of *σ_z_* predicted by Equation (1); and (**b**) the same data represented against the values of *q*, shown in Table 5, for different samples. Note that the first angular sample corresponds to the highest *q* and vice-versa.

In Figure 9, the same data are graphed differently. Figure 9a shows them cumulatively, together with the theoretical trend represented by Equation (1), evaluated for a hypothetical camera with *c* = 12 mm, at a distance of *h* = 200 mm from a hypothetical spherical object whose diameter was the average of all bounding box diagonals reported in Table 2 (i.e., *d* = 372.54 mm), rotating around its axis during the shooting. The sensor noise *σ_p_* was arbitrarily chosen in such a way to give a *σ_z_* equal to 250 μm, for the minimal baseline. 

The comparison shows qualitatively that except for the smallest baselines, the error tends to grow as the angular deviation grows, instead of decreasing, as theoretically expected. However, what appears clearly from Figure 8 and Figure 9a is that the behavior of the RMS error predicted by Equation (1) differs significantly from what is shown by the experiments.

As shown by the dashed line in shown in Figure 9a, where the distance h, the principal distance *c*, and the sensor (*σ_p_*) are always the same, Equation (1) predicts an indefinite decrease of the error with the growth of the angular displacement (i.e., the baseline), according to the hyperbolic trend, with the baseline *b* as the denominator. Contrarily, the experimental results in the same figure exhibit an initial decrease, reaching a minimal value, and an increase of the 3D uncertainty above a certain baseline.

From those experimental values, it is also possible to see how the object’s texture and reflective behavior affect the dense cloud uncertainty. We observe that specimen (a), in granite, with a nice grainy texture but slightly shiny, especially on the flat side, and specimen (d), in white marble, provide higher RMS errors than the opaque stone of samples (b) and (c), which are rich in recognizable features. In particular, the marble sample, having a noncooperative optical behavior, produces a trend different from all other materials—a circa-linear decrease of the error in the first three steps, followed by a circa-linear growth after the minimum.

Another element affecting the absolute RMS value is the ISO level. All samples (a−d) were captured at ISO 400, while specimen (e) at ISO 100. The results showed how this reduced the RMS by almost half of the material with the larger RMS at the first and last steps of the horizontal scale, while remaining below all other samples throughout the whole angular range. However, the trend of RMS vs. angle qualitatively followed the same behavior exhibited by the other samples captured with a higher ISO.

The trends reported in Figure 9b related to the same data shown in Figure 9a, where the horizontal scale was substituted with the parameter *q*, according to the correspondences with Δ*θ* reported in Table 5. Given that *q* was inversely proportional with the baseline *b*, and *b* was proportional to Δ*θ*, the samples represented by Figure 9b in correspondence with the smallest *q* correspond to those associated with the largest Δ*θ* in Figure 9a, and vice-versa. The purpose of this plot was to make those results comparable to other values reported in the literature like those summarized in Table 1.

Although the behaviors illustrated in both Figure 8 and Figure 9 make it possible to identify a consistent lowering of the RMS error from the second angular step, with a regrowth of the curves after the 4th step, a direct comparison among the different cases was not easy. This arises from the rather different absolute values of the errors determined by the different shape, texture, material–light interaction of the various samples, and ISO levels of the camera. 

We have, therefore, normalized all sequences referring to the minimum RMS error associated to the 8 sequences originated by each object, obtaining the values reported in Table 6.

**Figure 10:**
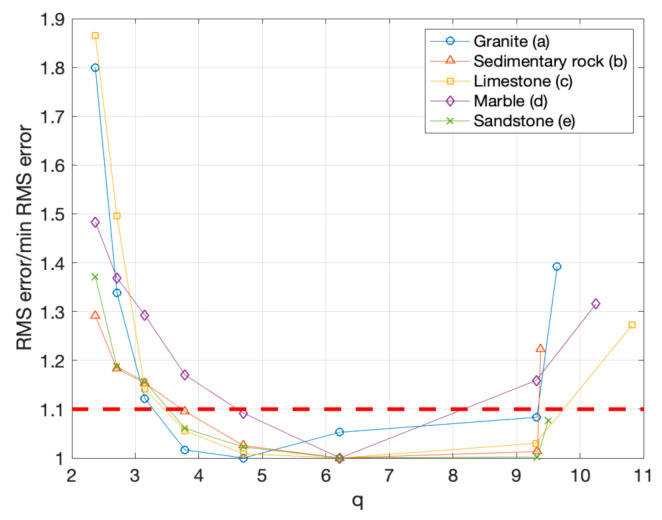
Normalized RMS errors for the different samples and different lateral displacements, also shown numerically in Table 6, plotted as function of q.

From this rearrangement of the RMS error, also shown in graphical form in Figure 10, it was possible to see the behavior more clearly. Each trend started with a non-minimal value at the minimal lateral displacement among photograms (the rightmost samples in the diagram of Figure 10), had a minimum after two or three angular steps (central area of the diagram), and tended to regrow for higher values of the angular step, namely for lower values of *q* (leftmost side of the diagram).

This behavior appeared to be consistent for all analyzed specimens, independent of size, texture, and material properties, with just minor behavioral differences, depending on the specific sample.

In particular, “Sample a” presents a minimal RMS error (normalized value = 1) for *q* = 4.7, while all the other samples had a minimal for *q* = 6.2.

**Figure 11:**
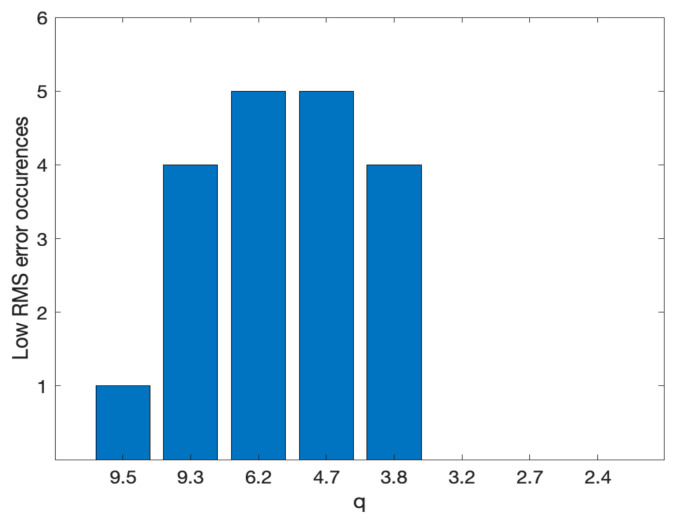
Histogram of occurrences where the RMS error is within a 10% tolerance from the minimum across the different test object used in the experiments. We can see that the large majority of “low RMS error” conditions corresponds to *q* = [3.8–9.3], while the range *q* = [4.7–6.8] identify the range where the point clouds of all five objects exhibit such low RMS error.

With the plot of Figure 11, we could also see for which *q* values the error remained below a predefined threshold represented by the dashed line, arbitrarily fixed at 10% above the minimal RMS error. The diagram indicated that most values below the threshold correspond to the central part of the diagram.

To give a more quantitative representation of this evidence resulting from the data, in Table 6, we also represented with a yellow background the “low error” situations, represented by values of the RMS error below the threshold. We can see that the errors within 10% of the minimum (i.e., normalized values < 1.1) tend to describe a pattern covering the first 5 lines of the table, corresponding approximately to values of *q*, ranging from 9.9 to 3.8.

In Figure 11, we quantify this result by representing as a histogram the number of occurrences of the “low error” condition across the different test objects, for the different values of q.

## 4. Discussion and Conclusions

This study analyzed the influence of the lateral displacement between adjacent images on the 3D quality of the dense cloud created by employing SfM/IM photogrammetry, in the case of highly convergent images, typical of close-range photogrammetry on objects.

To make this research comparable with textbooks and previous studies in the literature, the parameter through which we compared the different cases was *q*, the ratio between the average camera–target distance of two adjacent shots, and their mutual distance (baseline). As reported by several sources, this parameter was crucial in determining the quality of the 3D outcome of a photogrammetric measurement [19,21,22]. The value of *q* associated with preexisting research was then calculated for different published articles dealing with the influence of image overlap on the quality of aerial photogrammetry [35,36,37,38] and with micro-photogrammetry [39,40,41]. Even if not aimed at this study’s specific purpose, all mentioned studies contained clues indicating qualitatively that in SfM/IM photogrammetry, high values of *q* were preferable, even if values too high could be counterproductive.

Based on such observation, this study presented an experimental protocol and the related results for quantitatively exploring the influence of *q* over the quality of a 3D dense cloud generated by photogrammetry. The purpose was to spot a possible range of optimal *q* for maximizing the 3D quality photogrammetric outcome. Given the potential unmanageable number of experiments for analyzing any possible situation, the study was focused on a volume size typical of museum artifacts, using a photographic configuration broadly and successfully employed in several activities of massive 3D digitization of cultural heritage, using SfM/IM photogrammetry [10].

The study was carried out by analyzing several dense clouds obtained by different spatial distributions of cameras providing a considerable span of *q* [2.4–9.5]. The dense clouds were created by imaging five stone specimens, previously characterized with a high accuracy 3D active device and denoted as “a,” “b,” “c,” “d,” and “e.”

The experiments reported show that different from what is generally claimed for traditional photogrammetry, wherein the optimal *q* for convergent shooting in close-range photogrammetry lies in the range [0.4–0.8] [19], with SfM/IM close-range photogrammetry, such value grows significantly. In the experimental conditions described here, the results showed the lower RMS error at *q* = 4.7 for sample a, and at *q* = 6.2 for all other samples (b to e). The histogram represented in Figure 11 also showed that if we indicate as “low error” condition an increase of the RMS error within 10% of its minimum, the dense clouds generated photogrammetrically from all five samples satisfy the “low error” condition for *q* in the range [4.7–6.2], one order of magnitude larger than the values supposed to be optimal in traditional photogrammetry.

In our opinion, the reason for such a significant difference between the traditional and the SfM/IM-based photogrammetric error behavior lies in how the Image Matching phase affects the latter.

The typical calibration–resection–intersection process characteristic of photogrammetry is the same in the two cases.

Regarding the first two steps of calibration and resection (i.e., the camera’s orientation), the main difference lies in the correspondence identification shifting from operator-based in the former case to automatic in the latter, using feature extraction. The number of corresponding features passes in this way from tens/hundreds in the traditional approach to hundreds of thousands in SfM/IM photogrammetry. Therefore, when working on a textured natural scene, the bundle adjustment process that provides the self-calibration data and the camera positions becomes more robust in the automatic case than in the manual one.

However, while calibration and resection can provide better results with the automatic process, in the intersection phase, the situation is different. In traditional photogrammetry, each 3D point is extracted from images, through manual identification of the corresponding features, among oriented photograms by an operator. The selection of the points purposefully aimed only at those points that are considered to be useful for creating a 3D representation of the scene. The operator’s ability can affect the outcome, but this is not generally considered in any textbook, because it could not be generalized. Therefore, the sole geometrical factor was examined, keeping out human-based unpredictable elements.

In the automatic case considered here, the stereo image matching generated a dense cloud of points, by correlating an image patch from an oriented image with an array of pixels of another oriented image, aiming at the same area. This could more or less provide identifiable correlation peaks, depending on the object’s texture and superficial properties. Nevertheless, even in the presence of good texture, the correlation peaks would be less identifiable if the images were very far from each other, because perspective and illumination tend to modify the corresponding pixel contents. The possibility to find out false image-matching increases, which consequently increases the probability of wrong parallaxes originating from erratic behavior of the corresponding 3D points.

Even if in principle the results shown here should be valid, independent of the volume considered, this specific result gives practical support for defining a convergent cameras geometry for photogrammetric surveys of volumes, in the order of a museum object, whenever the critical feature to be pursued is the lowest measurement uncertainty (i.e., the best 3D quality).

In future work, we aim at better characterizing other parameters’ influence, as the ISO sensitivity, in correspondence to different textures, as well as exploring the effect of different image-matching algorithms on the presented issue. In particular, it would be interesting to explore the possible improvement in 3D quality vs. baseline, moving from a relatively basic stereo matcher as the one implemented in Agisoft Metashape, to a more sophisticated Multi-Image matcher.

## Figures and Tables

**Table 1:** Design factor *q* calculated for the different papers mentioned in this section with the overlap, sensor size, and focal length provided by the authors in the respective papers.

Paper	Sensor Size (mm × mm)	Focal Length (mm)	Distance (m)	Baseline (m)	Overlap	*q*
[35]	6.16 × 4.62	5.9	115	30	75%	3.84
[36]	6.16 × 4.62	4.0	80	4.9	96%	16.3
				9.8	92%	8.2
				14.7	88%	5.4
				19.6	84%	4.1
				24.5	80%	3.3
				29.4	76%	2.7
				34.3	72%	2.3
				39.2	68%	2.0
				44.1	64%	1.8
				49.1	60%	1.6
[37]	23.4 × 15.6	16	300	29.1	90%	10.3
				58.3	80%	7.5
				87.4	70%	3.3
				116.6	60%	0.8
[38] ^1^	6.16 × 4.62	4.0	1.2	0.069	95%	17.3
				0.208	85%	8.9
				0.346	75%	4.0
[40] ^2^	22.2 × 14.8	86.0	0.126	0.0055	-	22.9
				0.0220	-	5.7

^1^ For coherency with the other works, only the forward overlap was reported here, although the paper also discussed the lateral overlap and their combined effect on the photogrammetric results. ^2^ Baseline and *q* were calculated for ψ = 60° only, where the given depth of field had a lower effect on the final result [40].

**Table 2:** Bounding box sides and diagonals of each reference 3D mesh represented in Figure 4.

**Sample**	**x (mm)**	**y (mm)**	**z (mm)**	**d (mm)**
a	277.1	209.6	182.6	392.5
b	280.2	250.7	175.3	414.8
c	194.8	217.5	91.8	306.1
d	253.1	225.0	66.4	345.1
e	256.5	230.0	211.3	404.2

**Table 3:** Image sets processed for each object.

Set	Δ*θ* (rad)	Δ*θ* (°)	#Images
1	π/60	3	480
2	2π/60	6	240
3	3π/60	9	160
4	4π/60	12	120
5	5π/60	15	96
6	6π/60	18	80
7	7π/60	21	68
8	8π/60	24	60

**Table 4:** RMS error resulting from the comparison between the various point clouds originated by different angular displacements and the mesh reference, for the five test objects.

Δ*θ*	Sample a	Sample b	Sample c	Sample d	Sample e
(rad)	(μm)	(μm)	(μm)	(μm)	(μm)
π/60	242	190	207	241	136
2π/60	188	157	168	212	127
3π/60	183	155	163	183	126
4π/60	174	159	164	200	129
5π/60	177	170	172	214	134
6π/60	195	179	186	237	146
7π/60	233	184	244	251	150
8π/60	313	200	304	271	173

**Table 5:** Quality factor *q* calculated in correspondence of the different angular displacements (column 1) and the different object sizes (in parentheses below each sample identifier).

Δ*θ*	Sample a	Sample b	Sample c	Sample d	Sample e
(rad)	(393 mm)	(415 mm)	(306 mm)	(345 mm)	(404 mm)
π/60	9.64	9.38	10.82	10.25	9.50
2π/60	9.32	9.33	9.30	9.31	9.33
3π/60	6.22	6.22	6.22	6.22	6.22
4π/60	4.70	4.70	4.70	4.70	4.70
5π/60	3.78	3.78	3.78	3.78	3.78
6π/60	3.15	3.15	3.15	3.15	3.15
7π/60	2.71	2.71	2.71	2.71	2.71
8π/60	2.37	2.37	2.37	2.37	2.37

**Table 6:** Normalized RMS error with respect of the minimal value in the sequence associated with each object. The yellow values represent image configurations giving a normalized error lower than 1.1 (i.e., RMS error within 10% of the its minimal value in the sequence).

Δθ(rad)	q	Sample a	Sample b	Sample c	Sample d	Sample e
π/60	9.9 ^1^	1.392	1.223	1.272	1.315	1.077
2π/60	9.3	1.095	1.013	1.030	1.159	1.002
3π/60	6.2	1.042	1.000	1.000	1.000	1.000
4π/60	4.7	1.000	1.025	1.009	1.092	1.022
5π/60	3.8	1.081	1.096	1.056	1.170	1.061
6π/60	3.2	1.122	1.153	1.141	1.292	1.156
7π/60	2.7	1.154	1.183	1.496	1.369	1.187
8π/60	2.4	1.386	1.291	1.865	1.483	1.371

^1^ This value represents the average of the various values of *q* for the different samples at Δ*θ* = π/60 (first row of Table 5). The following *q* values are instead the same for all objects, if considered at the first decimal digit, and did not require any averaging.

## References

[1] Luhmann T. (2010). Close range photogrammetry for industrial applications. ISPRS J. Photogramm. Remote Sens..

[2] Taşçi L. (2015). Deformation monitoring in steel arch bridges through close-range photogrammetry and the finite element method. Exp. Tech..

[3] Iglhaut J., Cabo C., Puliti S., Piermattei L., O’Connor J., Rosette J. (2019). Structure from Motion Photogrammetry in Forestry: A Review. Curr. For. Rep..

[4] Villa C., Jacobsen C., Rutty G.N. (2019). The Application of Photogrammetry for Forensic 3D Recording of Crime Scenes, Evidence and People. Essentials of Autopsy Practice: Reviews, Updates and Advances.

[5] Ey-Chmielewska H., Chrusciel-Nogalska M., Fraczak B. (2015). Photogrammetry and Its Potential Application in Medical Science on the Basis of Selected Literature. Adv. Clin. Exp. Med..

[6] Evin A., Souter T., Hulme-Beaman A., Ameen C., Allen R., Viacava P., Larson G., Cucchi T., Dobney K. (2016). The use of close-range photogrammetry in zooarchaeology: Creating accurate 3D models of wolf crania to study dog domestication. J. Archaeol. Sci. Rep..

[7] Fau M., Cornette R., Houssaye A. (2016). Photogrammetry for 3D digitizing bones of mounted skeletons: Potential and limits. C. R. Palevol.

[8] Collet A., Chuang M., Sweeney P., Gillett D., Evseev D., Calabrese D., Hoppe H., Kirk A., Sullivan S. (2015). High-quality streamable free-viewpoint video. ACM Trans. Graph..

[9] Aicardi I., Chiabrando F., Lingua A., Noardo F. (2018). Recent trends in cultural heritage 3D survey: The photogrammetric computer vision approach. J. Cult. Herit..

[10] Guidi G., Malik U.S., Frischer B., Barandoni C., Paolucci F., Goodman L., Addison A. (2017). The Indiana University-Uffizi Project: Metrological Challenges and Workflow for Massive 3D digitization of Sculptures. Proceedings of the 2017 23rd International Conference on Virtual System and Multimedia (VSMM).

[11] Guidi G., Gonizzi Barsanti S., Micoli L.L., Russo M., Toniolo L., Boriani M., Guidi G. (2015). Massive 3D Digitization of Museum Contents. Built Heritage: Monitoring Conservation Management.

[12] Hess M., Robson S., Serpico M., Amati G., Pridden I., Nelson T. (2016). Developing 3D Imaging Programmes--Workflow and Quality Control. J. Comput. Cult. Herit..

[13] Santos P., Ritz M., Tausch R., Schmedt H., Monroy R., Stefano A.D., Posniak O., Fuhrmann C., Fellner D.W. CultLab3D—On the Verge of 3D Mass Digitization. Proceedings of the Eurographics Workshop on Graphics and Cultural Heritage.

[14] Gruen A., Remondino F., Zhang L. Image-based reconstruction of the Great Buddha of Bamiyan, Afghanistan. Proceedings of the SPIE, Videometrics VII.

[15] Guidi G., Russo M., Ercoli S., Remondino F., Rizzi A., Menna F. (2009). A Multi-Resolution Methodology for the 3D Modeling of Large and Complex Archeological Areas. Int. J. Archit. Comput..

[16] Georgopoulos A., Chanda B., Chaudhuri S., Chaudhury S. (2018). Contemporary Digital Technologies at the Service of Cultural Heritage. Heritage Preservation.

[17] Kasapakis V., Gavalas D., Dzardanova E., Lee N. (2018). 3D Modelling Through Photogrammetry in Cultural Heritage. Encyclopedia of Computer Graphics and Games.

[18] Driggers R.G. (2003). Encyclopedia of Optical Engineering.

[19] Luhmann T., Robson S., Kyle S., Boehm J. (2014). Close Range Photogrammetry: 3D Imaging Techniques.

[20] Sanz-Ablanedo E., Chandler J., Rodríguez-Pérez J., Ordóñez C. (2018). Accuracy of Unmanned Aerial Vehicle (UAV) and SfM Photogrammetry Survey as a Function of the Number and Location of Ground Control Points Used. Remote Sens..

[21] Waldhäusl P., Ogleby C.L. (1994). 3×3-rules for simple photogrammetric documentation of architecture. Proceedings of the ISPRS Commission V Symposium.

[22] Waldhäusl P., Ogleby C.L., Lerma J.L., Georgopolus A. 3 × 3 Rules for Simple Photogrammetric Documentation of Architecture. Updated Version 2013. https://www.cipaheritagedocumentation.org/wp-content/uploads/2017/02/CIPA__3x3_rules__20131018.pdf.

[23] Lowe D. (1999). Object Recognition from Local Scale-Invariant Features. IEEE Int. Conf. Comput. Vis..

[24] Bay H., Tuytelaars T., Van Gool L., Leonardis A., Bischof H., Pinz A. (2006). SURF: Speeded Up Robust Features. Computer Vision—ECCV 2006, Proceedings of the 9th European Conference on Computer Vision, Graz, Austria, 7–13 May 2006, Part I.

[25] Calonder M., Lepetit V., Strecha C., Fua P., Daniilidis K., Maragos P., Paragios N. (2010). BRIEF: Binary Robust Independent Elementary Features. Lecture Notes in Computer Science (Including Subseries Lecture Notes in Artificial Intelligence and Lecture Notes in Bioinformatics).

[26] Fischler M., Bolles R.C. (1981). Random sample consensus: A paradigm for model fitting with applications to image analysis and automated cartography. Commun. ACM.

[27] Torr P.H.S., Zisserman A. (2000). MLESAC: A New Robust Estimator with Application to Estimating Image Geometry. Comput. Vis. Image Underst..

[28] Zhou G., Wang Q., Xiao Z. (2017). Robust outlier removal using penalized linear regression in multiview geometry. Neurocomputing.

[29] Tang Z.-Z. (2012). Photogrammetry-based two-dimensional digital image correlation with nonperpendicular camera alignment. Opt. Eng..

[30] Tippetts B., Lee D.J., Lillywhite K., Archibald J. (2016). Review of stereo vision algorithms and their suitability for resource-limited systems. J. Real-Time Image Process..

[31] Furukawa Y., Ponce J. (2010). Accurate, Dense, and Robust Multiview Stereopsis. IEEE Trans. Pattern Anal. Mach. Intell..

[32] Hu X., Mordohai P. (2012). A Quantitative Evaluation of Confidence Measures for Stereo Vision. IEEE Trans. Pattern Anal. Mach. Intell..

[33] Wolf P.R., Dewitt B.A. (2000). Elements of Photogrammetry: With applications in GIS.

[34] Lemmens M. (2011). Photogrammetry: Geometric Data from Imagery. Geo-Information: Technologies, Applications and the Environment.

[35] Haala N., Rothermel M. (2012). Dense Multiple Stereo Matching of Highly Overlapping UAV Imagery. ISPRS.

[36] Dandois J., Olano M., Ellis E. (2015). Optimal Altitude, Overlap, and Weather Conditions for Computer Vision UAV Estimates of Forest Structure. Remote Sens..

[37] Ni W., Sun G., Pang Y., Zhang Z., Liu J., Yang A., Wang Y., Zhang D. (2018). Mapping Three-Dimensional Structures of Forest Canopy Using UAV Stereo Imagery: Evaluating Impacts of Forward Overlaps and Image Resolutions With LiDAR Data as Reference. IEEE J. Sel. Top. Appl. Earth Obs. Remote Sens..

[38] Zhou J., Fu X., Schumacher L., Zhou J. (2018). Evaluating Geometric Measurement Accuracy Based on 3D Reconstruction of Automated Imagery in a Greenhouse. Sensors.

[39] Sims-Waterhouse D., Isa M., Piano S., Leach R. (2020). Uncertainty model for a traceable stereo-photogrammetry system. Precis. Eng..

[40] Lavecchia F., Guerra M.G., Galantucci L.M. (2017). The influence of software algorithms on photogrammetric micro-feature measurement’s uncertainty. Int. J. Adv. Manuf. Technol..

[41] Galantucci L.M., Pesce M., Lavecchia F. (2016). A powerful scanning methodology for 3D measurements of small parts with complex surfaces and sub millimeter-sized features, based on close range photogrammetry. Precis. Eng..

[42] Guidi G., Micoli L.L., Gonizzi S., Brennan M., Frischer B. Image-based 3D capture of cultural heritage artifacts an experimental study about 3D data quality. Proceedings of the 2015 Digital Heritage International Congress.

[43] Godin G., Rioux M., Beraldin J.A., Levoy M., Cournoyer L., Blais F. An assessment of laser range measurement on marble surfaces. Proceedings of the 5th Conference on Optical 3D Measurement Techniques.

[44] Guidi G., Remondino F., Russo M., Spinetti A. Range sensors on marble surfaces: Quantitative evaluation of artifacts. Proceedings of the SPIE on Videometrics, Range Imaging, and Applications X.

[45] Guidi G. Metrological characterization of 3D imaging devices. Proceedings of the SPIE—The International Society for Optical Engineering.

[46] Evatronix Evixscan 3D Heavvy Duty Optima teChnical feAtures Sheet. https://evixscan3d.com/wp-content/uploads/2019/06/HDO_evixscan_3D_2019.pdf.

[47] Cignoni P., Callieri M., Corsini M., Dellepiane M., Ganovelli F., Ranzuglia G. MeshLab: An open-source mesh processing tool. Proceedings of the 6th Eurographics Italian Chapter Conference.

[48] Agisoft LCC Agisoft Metashape User Manual—Professional Edition, Version 1.6. https://www.agisoft.com/pdf/metashape-pro_1_6_en.pdf.

[49] Fraser C.S. (2013). Automatic Camera Calibration in Close Range Photogrammetry. Photogramm. Eng. Remote Sens..

